# Morphometric Study of the Intracranial Segment of the Vertebral Artery

**DOI:** 10.7759/cureus.22137

**Published:** 2022-02-11

**Authors:** Priya Dharshini, Gunapriya Raghunath, Karthikeyan Gurusamy, Zareena Begum, Savitha Dhamodaran, Balaji Karunakaran, Yuvaraj Maria Francis, Vandana Kaveripakkam

**Affiliations:** 1 Anatomy, Saveetha Institute of Medical and Technical Sciences, Chennai, IND; 2 Anatomy, Saveetha Medical College, Chennai, IND; 3 Anatomy, Tagore Medical College, Chennai, IND

**Keywords:** variation, vertebral artery, syncope, morphometry, computed tomography

## Abstract

Background

The vertebral artery (VA) forms the caudal part of the circle of Willis and is responsible for one-third of the blood supply of the cerebellum, pons, middle ear, and the upper part of the spinal cord and its meninges. The highest potential risk of injury to VA during cervical pedicle screw implantation is at C3 and then at C6. Knowledge about the morphometry of VA provides a better understanding of clinical conditions such as vertebra basilar insufficiency (VBI). Similarly, the knowledge of variation in the VA is needed during cervical pedicle screw implantation, which possesses the highest potential risk to VA at the C3 level. The origin of the vertebral artery from the aortic arch reached the upper cervical vertebra than the vertebral artery of subclavian origin. The length of the VA is greater on the left side than the right-side artery. Understanding and reporting of the same are essential to creating awareness that can aid in endarterectomy, angioplasty, and radiological procedures. Variations are noted in morphometry between sides.

Aim

To determine the morphometry of the vertebral artery (VA) with emphasis on the fourth segment (V4) and its variations using computed tomography angiogram (CTA).

Materials and methods

This present retrospective study was conducted in the Departments of Anatomy and Radiology and Imaging Sciences, Saveetha Medical College and Hospital. Participants were patients who took a head and neck CT for various clinical reasons. About 50 CT images, 33 male and 17 females, were selected from the archives. The length, diameter, and entry level of VA were studied. The data were tabulated and statistically analyzed.

Result

The mean length of VA was 24.49 ± 3.02 (cm) on the left side and 24.28 ± 3.91 (cm) on the right side in female subjects and was found to be 22.78 ± 1.7 (cm) on the left side and 21.5 ± 2.7 (cm) on the right side in male subjects. The mean diameter of VA at the level of the foramen magnum was 0.32 ± 0.05 (cm) on the right side and 0.322 ± 0.07 (cm) on the left side in females, 0.3 ± 0.064 (cm) on the left side and 0.26 ± 0.086 (cm) on the right side in males.

Conclusion

The length of VA was found to be statistically more on the left side than on the right side. The variations in morphometry seen can aid in various surgical and radiological procedures.

## Introduction

Syncope is the clinical manifestation in the posterior cerebral artery occlusion or temporary sludge flow in the vertebral artery (VA). VA stenosis is the primary risk factor for recurrent stroke [1]. All the vertebral arteries had a tortuous course and were covered with rich venous plexuses [2]. The variations in VA significantly alter the hemodynamics in the brain, leading to adverse effects [3]. The surgical approach in cervical spine surgery is either anterior or posterior [4]. The vertebral arteries (VA) arise from the first part of the subclavian arteries in the scaleno-vertebral triangle. Its course has been described in four parts, They are prevertebral (V1), vertebral (V2), atlantooccipital (V3), and intracranial (V4) segments [5]. The initial segment of the vertebral artery (V1) ascends to enter the transverse foramen (TF) of the six cervical vertebrae (V2). Then, it ascends through the transverse foramina of C6-C1, which runs posterolaterally around the atlas (V3). Finally, it penetrates the dura mater and passes through the foramen magnum to become the intracranial segment of the vertebral artery (V4) [6-7]. Both the VA unites together to form the basilar artery, which forms the caudal part of the circle of Willis to supply the hindbrain, which has the centers that control the respiratory and cardiac functions [8]. The vertebral artery is highly protected by bony and muscular structures in the neck, which makes it difficult to approach during surgical procedures. During cervical spine movements, VA undergoes large shear and tensile forces, elongated during lateral flexion, with kinking occurring during the rotation of the neck [9]. The aim of the study is to determine the variations and morphometry of the vertebral artery in both genders.

## Materials and methods

Study design

This retrospective study was done in the Department of Radiology and Imaging Sciences and Department of Anatomy, Saveetha Medical College and Hospital. The Institutional Review Board (IRB) of Saveetha Medical College approved the study and the IRB number for the study was SMC/IEC/2018/11/262.

Data collection

Fifty CT images, 33 male and 17 females, were selected from the archives of the Department of Radiology and Imaging Sciences, Saveetha Medical College and Hospital.

The parameters measured in the study were: 1. Length of VA (from origin to basilar artery) in cm; 2. The intracranial segment of VA - length in cm (from the foramen magnum to vertebrobasilar junction); 3. Diameter of the V4 segment of VA at the level of the foramen magnum (cm).

Inclusion criteria

CTA images from the archives of the Department of Radiology and Imaging Sciences, Saveetha Medical College and Hospital, were scanned. The age group chosen was between 16 and 70.

Exclusion criteria

These included: 1. Major cases showing stroke in CT; 2. Cases with developmental disorders of the central nervous system.

Statistical analysis

The length, diameter, and entry level of VA were studied. The data were tabulated and statistically analyzed. The parameters were measured by using the IntelliSpace Portal 9.0 software (Phillips, Amsterdam, Netherlands) to get the exact length of the artery.

The data were represented by mean ± SD and statistical analysis was done using the two-tailed student’s T-test in Microsoft Excel 2013 (Microsoft Corporation, Redmond, WA). P-value <0.05 was taken as statistically significant.

## Results

Origin of VA

The right vertebral artery had its origin from the subclavian artery and entered the foramen transversarium at the level of C6 in 44 cases (88%), whereas in six cases (12%), the left VA originated from the arch of the aorta and entered the foramen transversarium at the level of C5.

Length of VA

**Figure 1:**
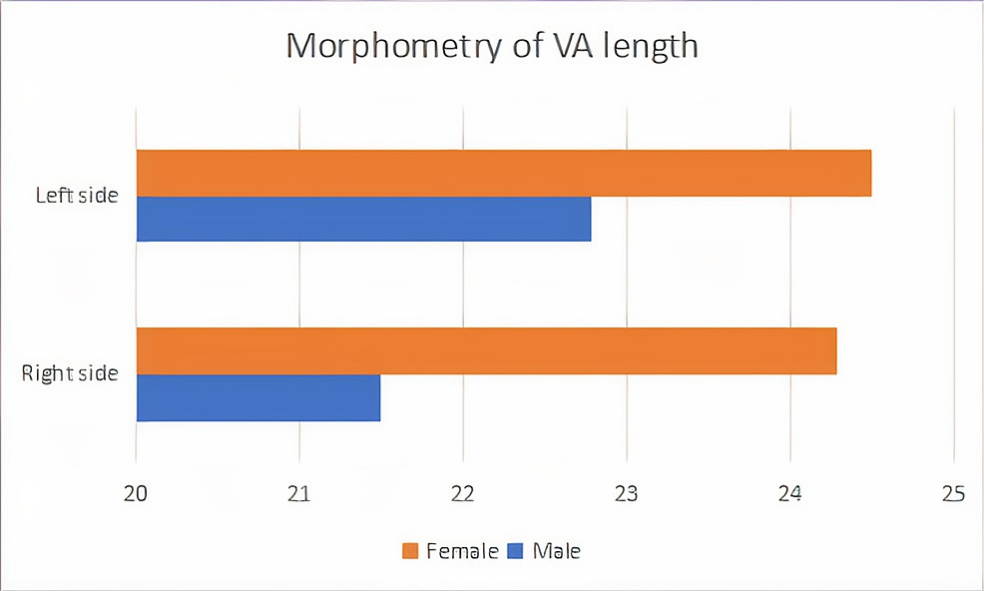
Representation of the length of VA of both sides in male and female subjects, where it is seen that in the majority of the subjects of both sexes, LVA showed greater length than RVA LVA - Left vertebral artery; RVA - Right vertebral artery

The length of VA was 24.49 ± 3.02 (cm) on the left side and 24.28 ± 3.9 (cm) on the right side in the female. The length of VA was 22.78 ± 1.7 (cm) on the left side and 21.5 ± 2.7 (cm) on the right side in male samples. Among the females, 61% showed an increase in the length of VA on the left than the right, 33% showed right-sided dominance, and 6% showed equal length on both sides. In the males, 84% showed an increase in the length of VA on the left side than the right side and 16% showed right dominance. The result showed left dominance in both genders. See Tables 1-2 and Figure 1.

**Table 1:** The length of vertebral artery (VA) as a whole and the further length and diameter of V4 at the foramen magnum level in both sexes The length and diameter of V4 showed statistical significance in male subjects. * Significant, ** Highly significant

Male	Right side (in cm)	Left side (in cm)	P-value
Length of VA	21.5±2.7	22.78±1.7	0.264294
Length of V4	3.23±0.97	3.80±0.7	0.041078 *
Diameter of V4 at foramen magnum	0.26±0.086	0.3±0.064	0.000499 **
Female	Right side (in cm)	Left side (in cm)	P-value
Length of VA	24.28 ±3.91	24.49 ± 3.02	0.862905
Length of V4	3.23±0.31	3.34±0.34	0.338541
Diameter of V4 at foramen magnum	0.32±0.05	0.32±0.07	0.915648

**Table 2:** The length variation of the left vertebral artery (LVA) and right vertebral artery (RVA) In female subjects, 61% showed longer LVA, 33% showed longer RVA, and 6% showed the same length. In male subjects, 84% showed longer LVA and 16% showed longer RVA. For V4 segment length, in female subjects, 71% showed longer LVA, 29% showed longer RVA, and in male subjects, 67% showed longer LVA and 27% showed longer RVA, with 2% showing similar length on both sides.

Length	% LVA > RVA	% RVA>LVA	% LVA=RVA
Female VA	61	33	6
Male VA	84	16	-
Female V4	71	29	-
Male V4	67	27	2

Length of the V4 segment

**Figure 2:**
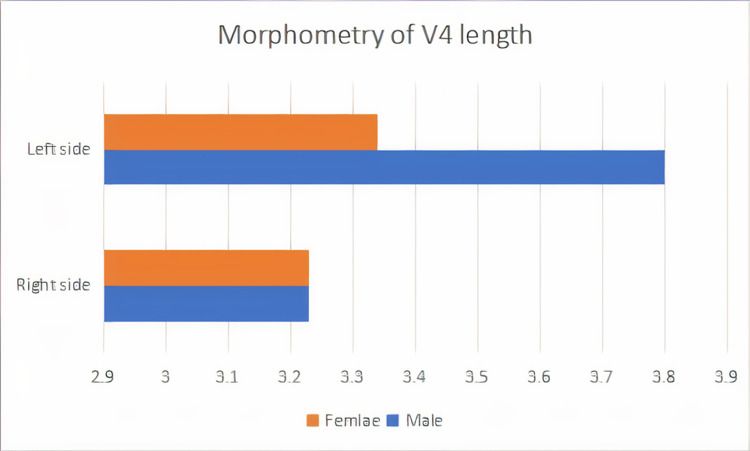
Representation of the length of the V4 segment of both sides in male and female subjects It is clearly seen that the left-sided V4 segment was found to be longer in males than in the female subjects, whereas right-sided data showed a similar length in both males and females. V4 - intracranial segment of the vertebral artery

The length of the V4 segment was 3.23 ± 0.31 (cm) on the right side and 3.34 ± 0.34 (cm) on the left side in females. In males, the length of the V4 segment was 3.23 ± 0.97 (cm) on the right side and 3.80 ± 0.7 (cm) on the left side. This study shows that 51% of the females have a longer V4 segment of VA on the left side than the right side and in the males, 54% of the cases have a longer V4 segment of VA on the left side than the right side. The left side V4 segment is longer than the right side (Figure 2).

Diameter of V4 segment at the level of the foramen magnum

**Figure 3:**
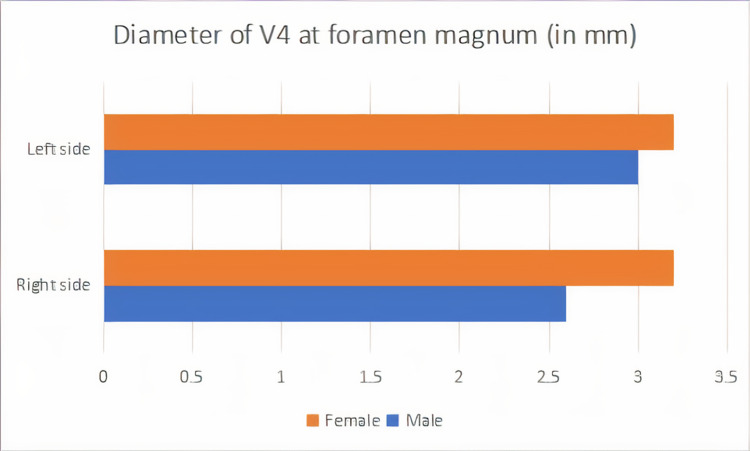
Representation of the diameter of the V4 segment at the level of the foramen magnum, where the male subjects showed greater diameter than female subjects on both sides V4 - intracranial segment of the vertebral artery

The diameter of the V4 segment at the level of the foramen magnum was 0.32 ± 0.05 (cm) on the right side and 0.32 ± 0.07 (cm) on the left side in the female. In males, the diameter was 0.3 ± 0.064 (cm) on the left side and 0.26 ± 0.086 (cm) on the right side. The present study shows that among female samples, the length of the V4 segment of VA was almost equal on both sides; among male samples, 57% showed dominance on the left side. See Figure 3.

Entry level of VA

In 88% of the cases, VA enters the TF at the C6 level, and in 12% of the cases, it enters at the C5 level.

## Discussion

**Figure 4:**
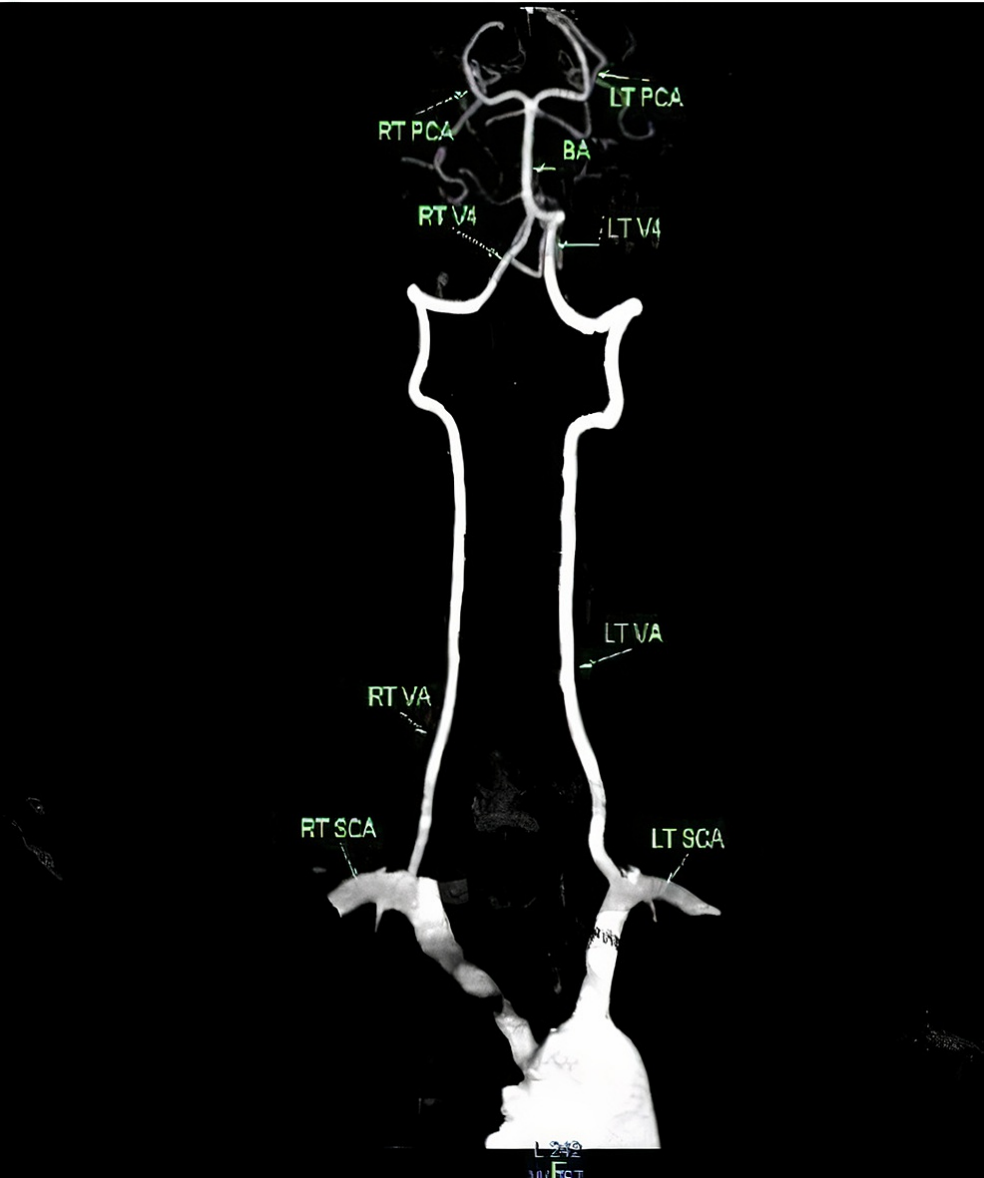
CT image showing the scheme of the vertebral artery as a whole CT - computed tomography

VA has a very unique, long, tortuous course through the neck and cranium [3]. One-quarter of strokes occur in the posterior circulation [10]. VA is continuously exposed to stress and strain. At the point of exit from the C2 vertebra, it is exposed to large shear and tensile forces during movements of the cervical spine. In spite of all these adverse conditions, damage to the artery is very less and the mechanism for protection lies in the structure of the arterial wall. According to most of the anatomical textbooks and researchers, the left VA is larger than the right [11]. Figure 4 is a CT image showing the scheme of the vertebral artery.

The results of VA origin were similar to the study conducted by Rawal et al. in 25 embalmed cadavers, who reported that from 50 dissected vertebral arteries, 46 vertebral arteries were found to enter the transverse foramen of the C6 vertebra (92%) and four vertebral arteries entered the transverse foramen of the C7 vertebra (8%) [11]. Bruneau et al. also described bilateral abnormality in 0.8% and unilateral abnormality in 12.4%, which was more common on the left side; they also described the vertebral artery entering the transverse foramen of C5 more often than the C7 vertebrae, which is similar to the present study [12]. These findings were also similar to the findings of Woraputtaporn et al., Wakalo et al., and Guilherme Dabus et al. (Table 3) [5,13-14].

**Table 3:** Comparison of available data on VA with other studies LVA - left vertebral artery; RVA - right vertebral artery; C4 - 4th cervical vertebra; C5 - 5th cervical vertebra; C6 - 6th cervical vertebra; C7 - 7th cervical vertebra

Entry level		C4	C5	C6	C7
Present study		-	12% (RVA)	88% (LVA)	-
Rawal et al. [11]		-	-	92% (LVA)	8%(RVA)
Bruneau et al. [12]		-	7%	90% (LVA)	3%
Hong JT et al. [15]		1.6%	3.3%	94.9%	-
Worawut et.al [5]	LVA	0.8%	4.2%	95%	-
	RVA	0.4%	-	99.6%	-

The morphological and structural characteristics of the intracranial part of the vertebral artery are becoming vital with the ever-increasing cases of cerebrovascular accidents. The anomalies of the vertebral artery are significantly critical, especially if there is an obstruction to other arteries supplying the brain. Vertebral artery hypoplasia may cause a reduction in the blood supply of the concerned region and give rise to neurological as well as vascular conditions like migraine, vestibular neuronitis, and medial and lateral medullary syndromes [3].

The significant part of the VA is the V4 segment, where aneurysms are common. Knowledge concerning anomalies and variations in the vertebrobasilar complex can be useful to radiologists in the diagnosis of associated aneurysms and in preventing complications during endovascular procedures. Hypoperfusion of brain tissues and hemodynamic insufficiency due to hypoplasia or stenosis of the VA is the basis for transient ischaemic stroke [16]. In the current study, the length varies on both sides, wherein the left side (53%) is longer than the right side (47%).

Ballesteros L et al. reported that the intracranial part of the VA has a mean total length of 33.86 mm on the left and 32.47 mm on the right [14]. The present study coincides with the findings of Ballesteros L et al., Akgun V et al., Songur et al and Tardieu et al. [17-20].

The length and diameter of the V4 were observed in the present study. There were no statistically significant differences in gender, however, the left artery was longer in both sexes, which was in concordance with a study done by Hong JM et al. [21]. However, a study done by Sureka B et al. showed a difference in the diameter of the male and female vertebral artery, where the female artery showed a smaller diameter as compared to male subjects [22]. The origin of the vertebral artery from the aortic arch reached the upper cervical vertebra than the vertebral artery of subclavian origin. The length of the VA is greater on the left side than on the right side. Understanding and reporting the same is essential to create awareness that can aid in various surgical and radiological procedures.

## Conclusions

This morphometric study on the vertebral artery showed that the length of the LVA is significantly different from RVA and that there is no gender difference. Similar findings were reported in the length of the V4 segment of VA where the difference in length of the left V4 segment and right V4 segment showed statistical significance. The entry point of the intracranial segment of VA showed different vertebral levels. Eighty-eight percent (88%) of the subjects showed the entry point of VA at the C6 vertebral level. These analyses and variations in morphometry may help vascular surgeons and radiologists avoid errors during diagnostic and treatment procedures.
